# The Impact of Endometriosis on the Quality of Life and the Incidence of Depression—A Cohort Study

**DOI:** 10.3390/ijerph17103641

**Published:** 2020-05-21

**Authors:** Damian Warzecha, Iwona Szymusik, Miroslaw Wielgos, Bronislawa Pietrzak

**Affiliations:** 1st Department of Obstetrics and Gynecology, Medical University of Warsaw, 02-015 Warsaw, Poland; damwarzecha@gmail.com (D.W.); miroslaw.wielgos@wum.edu.pl (M.W.); bronislawa.pietrzak@wum.edu.pl (B.P.)

**Keywords:** endometriosis, pelvic pain, quality of life, depression, infertility

## Abstract

The objective was to evaluate the quality of life and the incidence of depression among women suffering from endometriosis. Afterwards, the dependency between pelvic pain, its severity and stages of endometriosis were analyzed. The study protocol included women of reproductive ages with confirmed endometriosis. The stage of disease was conferred according to the ASRM (American Society of Reproductive Medicine) classification. Women fulfilled two questionnaires: “WERF EPHect Clinical Questionnaire” and self-prepared survey about fertility disorders. The study group comprised of 246 respondents. A total of 77.2% of women were symptomatic. The most common complaints were chronic pelvic pain (CPP, 71.1%), dysmenorrhea (69.0%) and dyspareunia (45.2%). Intensity of pain was independent from the stage of endometriosis. The incidence of infertility and the time to conceive increased with the stage of disease (stage 1—52.8%, 3.4 years; stage 2—66.7%, 4.1 years; stage 3—61.3%, 3.7 years; stage 4—96%, 6.1years; *p* = 0.02 and 0.03, respectively). The prevalence of depression was positively correlated with the beginning of dyspareunia (14.5 vs. 19.6 years old., *p* = 0.002). CPP (OR(odds ratio) = 3.8, 95% CI 1.2–12.8, *p* = 0.04) and painful defecation (OR = 7.7, 95% CI 1.4–42.3, *p* = 0.01) increased the risk of depression. Symptoms related to endometriosis and severity of pain correlate with the prevalence of depression. Stage of endometriosis is significantly related to the prevalence of infertility.

## 1. Introduction

Endometriosis is a chronic, inflammatory and disabling disease affecting many aspects of women’s lives. It is considered as one of the most common gynecological issues. According to epidemiological data, the incidence of endometriosis in the general population varies between 4% and 15%, depending on the source [1,2]. In some cases, endometriosis may have subclinical course; therefore, the real prevalence seems to be underestimated. Nevertheless, it was reported in up to 50% of women suffering from infertility [3]. Recent studies proved that endometriosis doubles the risk of infertility. The suspicion of the disease’s existence is usually based on its clinical manifestation. Affected women usually present pelvic pain, dysmenorrhea, dyspareunia and infertility during their reproductive years [4]. Visualization of endometriotic lesions upon surgery, with or without their biopsy, is considered as a gold standard in the diagnosis of the disease. The most useful clinical tool for staging of endometriosis is the classification introduced by American Society for Reproductive Medicine (ASRM) [5]. Unfortunately, these stages do not sufficiently correlate with its clinical manifestation [6].

Symptoms related to endometriosis itself, some treatment regimens and in some cases the need for surgical management negatively impact the quality of life. Patients with the symptomatic course of the disease present higher levels of stress, somatization and fatigue in daily life [7]. Previous studies also proved that those women more often suffer from depressive and anxiety disorders [8]. Fertility problems seem to be an independent risk factor for mental disorders. Decreased self-assessment, higher incidence of depressive symptoms and sexual dysfunctions are detected more often among couples suffering from infertility [9]. The ability to reproduce and be a parent is one of the fundamental needs in human life. According to the World Health Organization, infertility is defined as a failure to achieve pregnancy after 12 months of regular unprotected sexual intercourse [10]. The epidemiology of infertility depends on the geographic region, patient’s age or other comorbidities and ranges from 3.5% to 16.7% in developed countries [11]. This problem affects about 9% of Poles of reproductive age who aim to conceive [12]. Moreover, global trends in the prevalence of infertility had increased gradually from 42.0 million in 1990 to 48.5 million in 2010 [13]. Endometriosis is one of the conditions significantly reducing fertility rates due to multifactorial decline in the reproductive potential. Inappropriate cytokine secretion resulting in local and systemic inflammatory response, reduced ovarian reserve, decreased oocyte quality, altered embryo development or implantation failure are only a few possible mechanisms of infertility associated with endometriosis [14,15].

In the view of the aforementioned arguments, the destructive influence of both endometriosis and infertility on the quality of life is undeniable. Unexplained pathogenesis of endometriosis, lack of causal treatment and subclinical course of the disease in some patients indicate that more studies over these issues are urgently required.

The primary objective of the presented paper was to evaluate the quality of life and the incidence of depression among women suffering from endometriosis. The history of underlying disease reported symptoms and fertility impairments was taken into consideration as well. The secondary aim of the study was to analyze the dependency between the incidence of different kinds of pelvic pain, its severity and stages of endometriosis.

## 2. Materials and Methods

A single-center cohort study was carried out at the 1st Department of Obstetrics and Gynecology, Medical University of Warsaw. The study group consisted of patients with the diagnosis of endometriosis who attended the Outpatient Clinic between June 2017 and November 2019. The diagnosis of endometriosis was set in accordance with the guidelines of the European Society of Human Reproduction and Embryology (ESHRE), based on visual detection of endometriotic lesions during previous surgeries or ultrasonographic features of ovarian endometriomas only [16]. Data were obtained from individual medical records (not all the patients were operated on at our Department; however, their surgery protocols were thoroughly studied prior to inclusion). Patients with the suspicion of endometriosis, without surgical or ultrasound confirmation, were excluded from further analysis. The stage of endometriosis was conferred according to the revised American Society for Reproductive Medicine classification (rASRM) [5]. However, this staging is not a routine procedure at the time of surgery in all settings; therefore, not all the patients included in the study had an appropriate designation. The study protocol included only pre-menopausal women, aged 18–45 years, without any serious comorbidities that may reduce reproductive potential and adversely affect the quality of life (serious comorbidities included: multiple sclerosis, schizophrenia, severe anxiety disorders, type 1 diabetes mellitus and conditions causing physical disabilities, e.g., post-traumatic).

Women who met the inclusion criteria were asked to complete two questionnaires at the same time: modified “WERF EPHect Clinical Questionnaire” (The World Endometriosis Research Foundation, EPQ) including questions concerning the history and actual symptoms of endometriosis) and a self-prepared 16-item survey about fertility disorders [17]. Only women who fully completed both of the aforementioned forms were included in further analyses.

The EPQ form is freely available on the website of The World Endometriosis Research Foundation, a global charity focused on the problems and researches concerning endometriosis. The original 25-item English version of the questionnaire was translated into Polish by the authors of the presented paper. The utility of EPQ in order to acquire basic characteristics of patients with endometriosis was positively evaluated by previous investigators [18,19]. The survey concerns: general medical, obstetric and family history, as well as the clinical manifestations of endometriosis, especially the intensity and frequency of pelvic pain. The impact of reported symptoms on daily activities and the quality of life were taken into consideration as well. The intensity of pelvic pain and dysmenorrhea was assessed on 11-point Numeric Rating Scale (NRS), the most recommended pain scale for women with endometriosis [20]. The possible intensity of pain ranges from 0 (no pain) to 10 (worst possible pain). Further analyses were performed basing on the following cut-off points for mild (scores ≤5), moderate (6–7), and severe pain (scores ≥8) on the Numeric Rating Scale, due to the best model compliance between pain and functioning [21]. Chronic pelvic pain was defined as intermittent or constant pain in the lower abdomen or pelvis lasting for at least the last three months.

The self-prepared questionnaire created by the authors consisted of 16 questions concerning the history of previous fertility impairments, diagnostic tools and therapeutic procedures applied for the mentioned purpose. The final questions regarded the usage of contraceptive methods in the past, previous abdominal or pelvic surgery and other gynecological conditions that might be independent risk factors for fertility disorders.

The study was conducted with accordance to the Declaration of Helsinki for Medical Research involving Human Subject. Ethical approval was obtained from the Ethics Committee of Medical University of Warsaw (AKBE/99/2019). Each participant gave informed consent to the study.

### Statistical Analyses

Categorical variables were presented as percentages and continuous ones as means with standard deviations (SD). Baseline and clinical data were compared using nonparametric tests. Chi-square tests and ANOVA were performed. Univariate odds ratios (ORs) with 95% confidence intervals (95% CI) were calculated. Multiple logistic regression model was built to estimate which factors influence the severity of reported symptoms. Statistica 13.1 software (StatSoft Poland, Cracow, Poland) was used for statistical analyses. *p*-Values below the threshold of 0.05 were considered significant. Additional analyses included the adjustment for the stage of endometriosis and the duration of infertility.

## 3. Results

The study group comprised of 246 respondents. The baseline characteristics of women from the study group in relation to the stage of endometriosis are presented in Table 1.

All patients had endometriotic lesions confirmed during laparoscopy (64.8%), laparotomy (11.5%) or upon ultrasound examination (23.6%—typical ovarian cysts). A total of 75.2% of women underwent at least one laparoscopy (LPS) before, 21.2% at least two, while 6.8% more than two. The laparotomy (LPT) was performed significantly less frequently than LPS (*p* < 0.01): 35.8% of respondents had at least one open surgery before, 6% at least two, while 1.5% more than two. The stage of endometriosis was conferred according to ASRM classification in 111 respondents (45.1%). In multiple regression analysis, severe endometriosis (stages 3 and 4) was positively correlated with the incidence of infertility (OR = 15.8, 95% CI 1.9–132, *p* = 0.03); however, it was independent from patients’ age, age at menarche and current BMI. Neither the type of surgery (for LPS—*p* = 0.8, LPT—*p* = 0.6), nor the number of previous operations (*p* = 0.3 and 0.2, respectively) differed between subsequent stages of endometriosis.

Over half of the surveyed women (52.8%) had the diagnosis of infertility in the past. At the time of data collection, 95 respondents (39.7%) suffered from inability to conceive. There was significant difference in the incidence of infertility within stages of endometriosis (*p* = 0.02). This percentage increased with the severity of the disease. The mean time from of infertility to clinical pregnancy equaled 4.2 years (SD = 3.4). The time to conceive differed significantly among stages of endometriosis (stage 1—3.4 years (±2.3), stage 2—4.1 (±2.0), stage 3—3.7 (±3.9) and 6.1 (±4.9) years for stage 4, *p* = 0.03).

A total of 77.2% (183) of women reported any kind of symptoms related to endometriosis, as presented in Table 2. Only 22.8% (56) of respondents were asymptomatic. In the latter group, the diagnosis was usually set accidentally during surgery performed for other reasons. A total of 59.8% (147) of women had the diagnosis of endometrioma at the time of survey or in the past. The mean age at the diagnosis of ovarian cyst was 29.9 years (SD = 5.1). The incidence of the most frequently reported symptoms is presented in Table 2. The mean time frame between the diagnosis of endometriosis and the fulfillment of the questionnaire was 2.9 years (SD = 4.02). The most common types of pain in this group were chronic pelvic pain (71.1%), dysmenorrhea (69.0%) and dyspareunia (45.2%). Further sub-analysis assessing the risk of the aforementioned symptoms adjusted for the stage of endometriosis and parity revealed no significant differences regarding the above. The only feature that differed between consecutive stages of endometriosis was painful defecation (*p* = 0.04), which was positively correlated with the severity of endometriosis. 

The severity of particular types of pain adjusted for the stage of endometriosis are presented in Table 3. The intensity (from mild to severe) of dysmenorrhea, dyspareunia and pelvic pain were independent from the stage of endometriosis (*p* = 0.6, 0.5 and 0.15 respectively).

A total of 75.9% of women declared to use over-the-counter (OTC) analgesics during menstruation, while only 36.9% of them used painkillers prescribed by a doctor. The need for analgesics was independent from the stage of endometriosis (*p* = 0.9). A total of 31.7% of study participants used oral contraceptives at the time of data collection.

A total of 39.9% of surveyed women suffering from endometriosis admitted that they had visited the Emergency Unit due to severe menstrual pain at least once in the past. More than half of the respondents declared that pelvic pain reduced their physical, sexual and professional activity within the preceding 3 months (70.2%, 69.5% and 39.5%, respectively).

A total of 15.1% of women with endometriosis had the diagnosis of depression. The mean age at the onset of depressive symptoms was 22.2 (SD = 7.6) years. The prevalence of depression at the time of data collection was positively correlated with the age of the onset of dyspareunia (14.5 years of age, SD = 4.3 vs. 19.6 SD = 7.4 in the group without depression, *p* = 0.002). The incidence of depressive symptoms or chronic fatigue was independent from the stage of endometriosis (*p* = 0.8 to 0.9 for each stage). In multiple regression analysis, of all reported types of pain, only chronic pelvic pain and painful defecation reached significant influence on the incidence of depressive symptoms (Table 4).

Moreover, dysmenorrhea and pelvic pain significantly reduced physical activity within last three months (OR = 3.5, 95% CI 1.4–8.7 and OR = 3.5, 95% CI 1.3–8.9, respectively). Neither the diagnosis of infertility nor the duration of treatment were related to the incidence of depression (OR = 0.7 95% CI 0.4–1.4 and OR = 0.9 95% CI 0.8–1.2). 

## 4. Discussion

The dependency between endometriosis and different kinds of pelvic pain is well established; however, strict mechanisms involved in its pathogenesis are still under investigation. The presented paper reports that the most common symptoms among affected women are chronic pelvic pain (71.1%), dysmenorrhea (69.0%), back pain (54.0%), dyspareunia (45.2%) and painful defecation (36.5), while 22.8% of women diagnosed with endometriosis are asymptomatic. The obtained results are in line with the previous literature reports [22,23]. A large case-control study carried out in the United Kingdom of over 5500 women with endometriosis reported 73 percent incidence of abdominopelvic pain or dysmenorrhea compared with 20 percent of not affected controls [22]. Another cohort study including over 600 women with endometriosis identified seven symptoms associated with the disease that included abdominal pain with no relation to menstruation, pain during urination, pain during defecation, constipation or diarrhea, irregular bleeding, nausea or vomiting and feeling tired or lacking energy [24]. According to the literature, factors associated with an increased risk of endometriosis include age, nulliparity, early menarche and low BMI [22,25]. The authors of the presented paper did not find any significant correlation between aforementioned variables and the stage of the disease. Moreover, no correlation between the most frequently reported symptoms (dysmenorrhea, dyspareunia and pelvic pain) and the stage of the underlying disease was found. Painful defecation was the only feature that differed between consecutive groups, and its incidence was positively correlated with the severity of endometriosis. Painful defecation may be a result of large, deep infiltrating endometriotic lesions in the pouch of Douglas and rectovaginal septum. This is a probable reason why the above symptom was more often reported in advanced stages of the disease. According to all of the above results, symptoms related to endometriosis poorly predict its stage. Acquired data are in accordance with other researchers who reported similar pain characteristics, and no clear correlation between the stage of endometriosis and occurrence or severity of pain symptoms [6,26,27]. Only Vercellini et al. observed poor correlation between the stage of the disease and the severity of dysmenorrhea and non-menstrual pain (OR = 1.33, 95% CI = 1.04–1.71 and 1.01, 95% CI = 1.00–1.03, respectively). The above Italian study covering over 469 women suffering from endometriosis also revealed no clear-cut association between the stage, site or morphological characteristics of pelvic endometriosis and different types of pain [6]. 

It is hard to extrapolate the findings concerning the incidence of different types of pain and related symptoms into the general population because the study did not include the control group. However, it was intentional, to analyze highly selected group of patients for whom WERF EPHect Clinical Questionnaire was dedicated and validated. It is difficult to exclude other potential causes of pelvic pain or dysmenorrhea and reach the homogenous group of controls. Moreover, even in the group of healthy patients, a certain percentage of undiagnosed endometriosis should be expected. Questions regarding disabilities or other chronic conditions that may themselves decrease the quality of life were included in the questionnaire. As pointed out in material and methods section, all patients with severe comorbidities were excluded from further analyses to reduce the risk of bias. Nevertheless, some psychological/psychiatric conditions, such as untreated mild anxiety disorders, might have been present in our study group. Due to a relatively short time between the diagnosis of endometriosis and the fulfillment of the questionnaire, we assume that there is a low risk of possible progression of endometriosis from the diagnosis until the time of survey.

A total of 15.1% of women with endometriosis in the study group had been diagnosed with depression, similarly to the findings presented by Fried et al. who reported 14.5% incidence of depressive and 29% of anxiety symptoms among Austrian subjects suffering from endometriosis [28]. Recent metanalysis carried out by Gambadauro et al. (24 studies, 99,614 women) proved increased levels of depression in this group of patients (standardized mean difference of 0.49) [29]. Furthermore, women with endometriosis and pelvic pain seem not to have higher incidence of depressive symptoms compared to those with pelvic pain without endometriosis. On the other hand, endometriosis with accompanying pelvic pain increases the rate of depressive symptoms more significantly than endometriosis without pain. This data suggest that pain may be a greater determinant of depressive symptoms than the disease alone. The presented paper showed that the mean age at the onset of depressive symptoms was 22.2 years, close to the onset of symptoms related to endometriosis itself (18.8 to 24 years). Moreover, of all reported types of pain, chronic pelvic pain and painful defecation significantly enhanced the incidence of depressive symptoms (OR = 3.8 and 7.7, respectively). The risk of depressive symptoms or chronic fatigue was independent from the stage of the disease. The authors of the study found only two other researches evaluating similar dependency. Both of them reported no correlation between psychiatric symptoms and the stage of endometriosis; however, the study groups were limited to only 68 and 104 participants [30,31].

The destructive impact of endometriosis on the quality of life indicates significantly reduced physical and sexual activity among affected women, due to persistent dysmenorrhea and pelvic pain. According to the authoritative guidelines, early screening for depressive symptoms and multidisciplinary approach in diagnosed patients are highly advised [16,32]. In this view, clinicians who take care of women suffering from chronic pelvic pain, especially coexisting with endometriosis, should be aware of an increased risk of depressive disorders.

According to other previous researches, the greatest prevalence of endometriosis was observed between 25 and 29 years of age [33]. However, several studies have reported large diagnostic delay with an average time from the onset of first symptoms to the final diagnosis from 4.4 years in USA to 10.4 years in Germany [34,35]. Hypothesized main reasons for such a great delay include intermittent use of contraceptives, misdiagnosis and self-treatment of pain with OTC painkillers. These findings are consistent with the outcomes of the presented study concerning the mean age at the recognition of the disease (26.9 years old) and the onset of symptoms related to endometriosis (form 18.8 years of age for dysmenorrhea to 24.0 for dyspareunia).

Of all considered baseline characteristics, only the incidence of infertility significantly increased with the severity of the disease (*p* = 0.02). Severe endometriosis (stages 3 and 4) was positively correlated with the incidence of infertility (OR = 15.8). The mean time from the diagnosis to clinical pregnancy equaled 4.2 years and extended throughout subsequent stages of endometriosis. Due to the reduced fertility rates and prolonged conception awaiting period, women with endometriosis, especially in stages 3 and 4, should be referred to the reproductive medicine specialist as soon as they decide to get pregnant. These findings stay in accordance with the recommendations of the European Society of Human Reproduction and Embryology [16]. A large epidemiological study carried out by Saraswat et al. showed that endometriosis extends the conception awaiting period (mean time from the diagnosis to pregnancy: 2 years and 4 months) [36]. Moreover, pregnant women with endometriosis tend to be older than unaffected ones (30.5 vs. 27.2). Unambiguous correlation between the severity of endometriosis and the prognosis for fertility treatments had been reported before. D’Hooghe et al. indicated an increased incidence of subfertility in patients with more advanced stages of endometriosis [37]. Harb et al. proved that there is a less negative influence of minimal and mild endometriosis on reproductive outcomes in both spontaneous conception and assisted reproductive techniques (ART) [38]. Nevertheless, more recent studies showed that ASRM classification poorly correlates with pregnancy rates. According to them a new staging system, the endometriosis fertility index (EFI), should be introduced to counsel patients on their fertility management options [39,40]. To the authors’ knowledge, this staging system is still not widely utilized in infertility centers in Poland. 

ASRM Practice Committee Recommendations concerning the treatment of pelvic pain associated with endometriosis, advise “lifelong management plan with the goal of maximizing the use of medical treatment and avoiding repeated surgical procedures” [41]. Patients from the presented study group more often underwent less invasive procedures in the past (LPS vs. LPT). Repeated surgical treatment increases a potential risk of diminished ovarian reserve, which may negatively impact further fertility [42]. The analysis of previous surgeries performed in the study group showed a relatively high rate of patients who required repeated operative treatment. A total of 21.2% of women underwent more than one LPS, while 6.8% at least three. The incidence of repeated open surgeries was significantly lower and equaled 6% and 1.5%, respectively. The stage of endometriosis did not influence the kind and number of previous surgeries. According to these findings, the authors recommend the usage of less invasive operative techniques. Laparoscopy seems to be efficient and relatively safe technique in diagnosis and treatment of endometriosis, which is in line with previous investigations [43,44].

A total of 75.9% of respondents in the above research declared to use over-the-counter analgesics during menstruation, while only 36.9% of them used painkillers prescribed by a doctor. Similar findings were obtained in females with primary dysmenorrhea. In this group, up to 70% of participants practiced self-management with OTC painkillers, while only 15% used a prescription medication [45,46]. The need for analgesics was independent from the stage of endometriosis. These data are in line with previous conclusions regarding the lack of correlation between the severity of pelvic pain and the stage of endometriosis.

## 5. Conclusions

Endometriosis related pain, but not the disease itself, seems to increase the prevalence of depression. Reduced physical and sexual activity due to the symptoms related to the underlying disease are observed in the majority of affected women. On the basis of the available evidence, there is no significant correlation between the severity of endometriosis and the incidence of different types of pain. Therefore, symptoms related to endometriosis poorly predict its stage. However, the stage of endometriosis is significantly related to the prevalence of infertility, which seems not to correlate with the incidence of depression in those women. Further studies investigating the pathophysiology between endometriosis and depression or fertility impairments are required.

## Figures and Tables

**Table 1:** Baseline characteristics of the study group.

Variable	Total	Stage 1	Stage 2	Stage 3	Stage 4	*p*-Value
Sample size	246	27 (24.3%)	27 (24.3%)	31 (27.9%)	26 (23.4%)	N/A
Age (years)	33.5 (±6.6)	30.6 (±4.7)	30.4 (±4.1)	31.4 (±4.7)	32.7 (±4.0)	0.1
Age at menarche (y.o.)	12.8 (±1.5)	12.6 (±1.3)	12.5 (±1.4)	12.8 (±1.8)	12.6 (±1.7)	0.7
Age at diagnosis (y.o.)	29.9 (±5.1)	25.7 (±3.9)	24.3 (±5.2)	27.3 (±4.3)	24.1 (±4.9)	0.9
BMI (kg/m^2^)	23.2 (±4.1)	23.6 (±3.5)	21.5 (±2.8)	25.0 (±5.2)	23.1 (±3.9)	0.1
Nulliparity	152 (61.8%)	12 (44.4%)	14 (51.8%)	13 (41.9%)	19 (73.1%)	0.09
Infertility	130 (52.8%)	18 (66.7%)	20 (74.1%)	19 (61.3%)	25 (96.1%)	0.02
Tobacco users	37 (15.0%)	7.4%	22.2%	19.4%	23.1%	0.4

±—Standard deviation; BMI—body mass index; y.o.—years old; N/A—not applicable.

**Table 2:** The incidence of different types of pain within patients with endometriosis.

Symptoms	Incidence *n* (%)	Age of Onset y.o. (SD)	Stage of Endometriosis				
			1—*n* (%)	2—*n* (%)	3—*n* (%)	4—*n* (%)	*p*-Value
Pelvic pain	170 (71.1)	22.1 (7.8)	13 (48.1)	16 (59.3)	24 (77.4)	19 (73.1)	0.5
Dysmenorrhea	165 (69.0)	18.8 (7.2)	18 (66.7)	20 (74.1)	25 (80.6)	21 (80.8)	0.6
Dyspareunia	108 (45.2)	24.0 (6.7)	13 (48.1)	15 (55.6)	16 (51.6)	14 (53.8)	0.9
Painful defecation	87 (36.5)	23.9 (6.6)	7 (25.9)	13 (48.1)	19 (61.3)	15 (57.7)	0.04
Blood in the stool	13 (5.4)	n/a	3 (11.1)	0	3 (9.7%)	4 (15.4)	0.2
Painful micturition	31 (13.0)	26.2 (4.2)	5 (18.5)	4 (14.8)	4 (12.9)	4 (15.4)	0.9
Blood in the urine	7 (2.9)	n/a	1 (3.7)	0	0	1 (3.8)	0.8
Back pain	129 (54.0)	n/a	11 (40.7)	15 (55.6)	20 (64.5)	17 (65.4)	0.2
Fatigue	111 (46.4)	n/a	11 (40.7)	10 (37.0)	17 (54.8)	16 (61.5)	0.2

SD—Standard Deviation; y.o.—years old.

**Table 3:** Severity of pain adjusted for the stage of endometriosis.

Symptoms	Mean NRS (SD)	Stage 1 NRS (SD)	Stage 2 NRS (SD)	Stage 3 NRS (SD)	Stage 4 NRS (SD)	*p*-Value
Pelvic pain	4.7 (2.6)	4.7 (1.6)	5.5 (2.4)	5.6 (2.2)	5.3 (2.4)	0.2
Dysmenorrhea	6.1 (2.4)	7.3 (1.8)	5.4 (2.6)	6.7 (2.2)	6.4 (2.0)	0.3
Dyspareunia	4.5 (2.2)	4.8 (2.2)	4.9 (2.9)	5.1 (2.2)	4.4 (1.9)	0.8

NRS—Numeric Rating Scale; SD—Standard Deviation.

**Table 4:** Multiple regression analysis. Different types of pain and the diagnosis of infertility in relation to the prevalence of depression.

Type of Pain	OR	95% CI	*p*-Value
Chronic pelvic pain	3.8	1.2–12.8	0.04
Dysmenorrhea	0.8	0.3–2.6	0.74
Painful defecation	7.7	1.4–42.3	0.01
Dyspareunia	1.3	0.6–3.1	0.52
Painful micturition	0.5	0.2–1.6	0.26
Infertility in the past	0.8	0.3–2.7	0.8
Current infertility	1.0	0.3–3.7	0.9

CI—Confidence Interval; OR—Odds Ratio.
